# CT Angiography Manual Multiplanar Vessel Diameter Measurement vs. Semiautomated Perpendicular Area Minimal Caliber Computation of Internal Carotid Artery Stenosis

**DOI:** 10.3389/fcvm.2021.740237

**Published:** 2021-12-09

**Authors:** Timo Siepmann, Kristian Barlinn, Thomas Floegel, Jessica Barlinn, Lars-Peder Pallesen, Volker Puetz, Hagen H. Kitzler

**Affiliations:** ^1^Department of Neurology, University Hospital Carl Gustav Carus, Technische Universität Dresden, Dresden, Germany; ^2^Institute of Diagnostic and Interventional Neuroradiology, University Hospital Carl Gustav Carus, Technische Universität Dresden, Dresden, Germany

**Keywords:** vessel, ICA, imaging, algorithm, stenosis, neurovascular, hemodynamics, neuroradiology

## Abstract

**Objective:** To determine the diagnostic agreement of CT angiography (CTA) manual multiplanar reformatting (MPR) stenosis diameter measurement and semiautomated perpendicular stenosis area minimal caliber computation of extracranial internal carotid artery (ICA) stenosis.

**Methods:** We analyzed acute cerebral ischemia CTA at our tertiary stroke center in a 12-month period. Prospective NASCET-type stenosis grading for each ICA was independently performed using (1) MPR to manually determine diameters and (2) perpendicular stenosis area with minimal caliber semiautomated computation to grade luminal constriction. Corresponding to clinically relevant NASCET strata, results were grouped into severity ranges: normal, 1–49%, 50–69%, and 70–99%, and occlusion.

**Results:** We included 647 ICA pairs from 330 patients (median age of 74 [66–80, IQR]; 38–92 years; 58% men; median NIHSS 4 [1–9, IQR]). MPR diameter and semiautomated caliber measurements resulted in stenosis grades of 0–49% in 143 vs. 93, 50–69% in 29 vs. 27, 70–99% in 6 vs. 14, and occlusion in 34 vs. 34 ICAs (*p* = 0.003), respectively. We found excellent reliability between repeated manual CTA assessments of one expert reader (ICC = 0.997; 95% CI, 0.993–0.999) and assessments of two expert readers (ICC = 0.972; 95% CI, 0.936–0.988). For the semiautomated vessel analysis software, both intrarater reliability and interrater reliability were similarly strong (ICC = 0.981; 95% CI, 0.952–0.992 and ICC = 0.745; 95% CI, 0.486–0.883, respectively). However, Bland–Altman analysis revealed a mean difference of 1.6% between the methods within disease range with wide 95% limits of agreement (−16.7–19.8%). This interval even increased with exclusively considered vessel pairs of stenosis ≥1% (mean 5.3%; −24.1–34.7%) or symptomatic stenosis ≥50% (mean 0.1%; −25.7–26.0%).

**Conclusion:** Our findings suggest that MPR-based diameter measurement and the semiautomated perpendicular area minimal caliber computation methods cannot be used interchangeably for the quantification of ICA steno-occlusive disease.

## Introduction

Accurate quantification of the degree of the extracranial internal carotid artery (ICA) stenosis is pivotal in determining the optimal treatment regimen because the risk of stroke and the benefit from surgical treatment *via* carotid endarterectomy increase with the degree of stenosis (1). Digital subtraction angiography shows excellent precision in the quantitative measurement of ICA stenosis but is limited by invasiveness and increased duration of the procedure when compared with non-invasive imaging techniques, such as duplex sonography or computed tomography angiography (CTA) (2). This is particularly important in the initial evaluation of patients with acute cerebral ischemia, in whom time to initiation of thrombolytic or endovascular recanalization treatment is a major predictor of clinical outcome and each minute in which stroke remains untreated results in significant loss of central nervous system neurons (3, 4). In patients presenting with symptoms of acute cerebral ischemia, CTA-based analysis of ICA luminal constriction using the North American Symptomatic Carotid Endarterectomy Trial (NASCET) criteria provide the most time effective and feasible way to quantify ICA stenosis with high sensitivity and high negative predictive value for steno-occlusive disease (5). Since NASCET grading is commonly performed manually, the level of experience of the respective rater potentially biases grading of this manual assessment (1, 6). Further standard CTA contrasted vessel top-view diameter measurements neglect the potentially intraluminal stenosis configuration affected by non-circular plaque surface and irregular calcification formation potentially resulting in an underestimation of stenosis assessment. To address this issue of potential error, semiautomated software-based ICA constriction measurement and stenosis assessment procedures were introduced (7).

Although several studies have supported the capacity of these techniques to accelerate measurement procedure times and reduce interobserver variability, translation into clinical practice has been limited and the optimal technique has still to be determined (7–11). Generally, these semi automated methods provide the determination of the perpendicular endoluminal stenosis area. Using the simultaneously computed minimal caliber, this approach seems to be a more precise foundation for quantifying the degree of stenosis based on irregular plaque geometry considerations. Viability and utility of using the computed stenosis perpendicular area minimal caliber as the primary method of extracranial ICA stenosis grading in acute cerebral ischemia workup are yet unknown (12).

We (1) synchronously performed standard CTA manual multiplanar reformatting (MPR)-based stenosis diameter measurement and the semiautomated perpendicular stenosis area minimal caliber computation and used both luminal constriction measures for extracranial ICA stenosis grading in initial diagnostic CTA acquired in patients with suspected cerebral ischemia and explored the advanced technique's feasibility in the acute ischemia workup. We further (2) tested the hypothesis that ICA stenosis quantification by means of perpendicular area minimal caliber computation coincides with the established manual MPR diameter measurements in regard to the NASCET-type stenosis categories using adaptive grading comparison.

## Materials and Methods

### Study Population

This study analyzed consecutive patients with acute ischemic stroke or transient ischemic attack who were admitted to our tertiary stroke center over a 12-month period. Patients were eligible for inclusion if their diagnostic workup included CTA. Demographic characteristics and baseline stroke severity with the National Institutes Health Stroke Scale (NIHSS) score were collected.

### Computed Tomography Angiography

A 64-slice CT scanner (SOMATOM Sensation 64, Siemens Healthineers, Erlangen, Germany) was used. Multislice CT acquisition was performed using isotropically resolved contrast media-enhanced angiographic imaging of extracranial vessels. To achieve optimal timing of arterial contrast bolus tracking, a region of interest within the aortic arch and a threshold set to 120 Hounsfield units (HU) were used. The procedure utilized 80 cc intravenous contrast of Solutrast® 370 (Bracco Imaging Deutschland GmbH, Konstanz, Germany) or Ultravist® 370 (Schering/Bayer Pharma AG, Berlin, Germany) with an injection rate of 3–4 cc/s followed by 50 cc sodium chloride injection. Other parameters were as follows: 100 KV, effective 160 mAs, rotation time 0.5 s, detector collimation 0.6 mm, reconstructed slice thickness 0.75 mm, pitch 1.2, kernel H20, and image acquisition order caudal cranial.

### MPR-Based Manual ICA Stenosis Diameter Measurements

**Figure 1:**
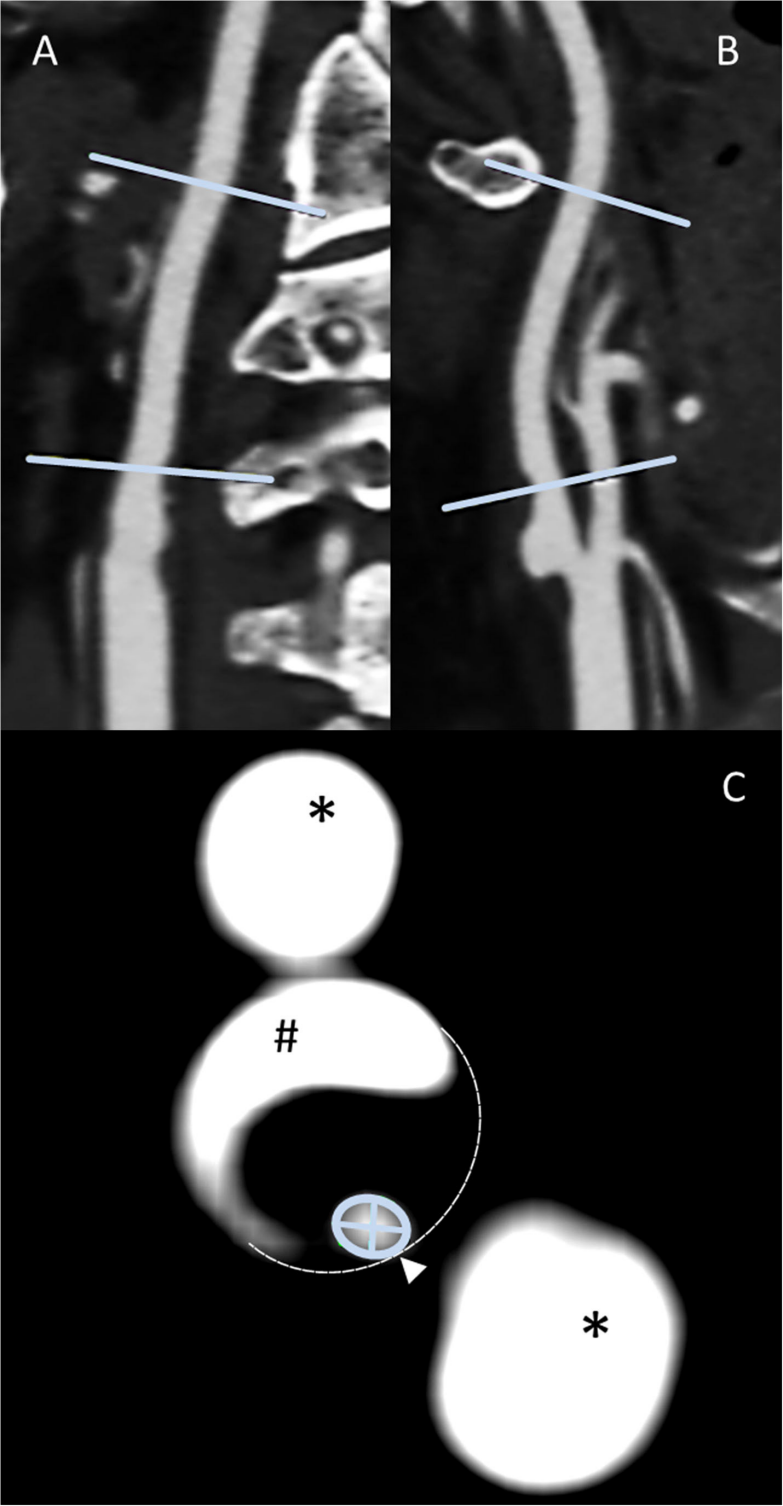
Methodology panel, MPR-based ICA stenosis diameter measurements: sagittal **(A)** and coronal **(B)** multiplanar reconstructions allow the identification of both the non-stenotic distal diameter [NASCET **(C)**] and the stenosis region. After the adjustment of either the coronal or sagittal plane for the optimal perception of the stenotic vessel configuration along the stenotic segment principal orientation, the subsequent perpendicular axial reconstruction of excellent spatial CTA resolution allows the outstanding distinction of vascular pathology **(C)**, that is, calcified plaque (#), non-contrasted soft plaque (area enclosed by dashed line), and residual ICA lumen (encircled by green line). This plane is used for locating the smallest diameter [NASCET **(A)**]. Finally, the minimum stenotic diameter can be measured defined as the smaller of the two measurable diameters. External carotid artery branches (*) appear as homogenously contrasted additional lumina.

All manual image analyses were prospectively treated and analyzed by a physician with expertise in cerebrovascular imaging blinded to clinical and also any other imaging findings and outcomes. All quantitative measurements were supervised by an expert neuroradiologist (HHK). Obtained CTA raw data were initially screened for overall quality, carotid artery occlusion, and the presence of bifurcation calcifications. Initially, maximum intensity projection (MIP) allowed the exploration of vascular anatomy by increasing the artery-to-tissue contrast to define the presence and location of the ICA stenosis. Subsequently, the dataset was reformatted using MPR to generate consecutive and freely adjusted planes in respect of the ICA orientation allowing for precise measurement of the extent of the stenosis by minimal diameter measurements. Whereas, two MPR planes were set along the principal artery axis, the third was adjusted orthogonally to both planes and adapted in the presence of irregular stenosis. The standard Hounsfield scale center and window (c/w) were set to 250/600. In the presence of calcifications, c/w was adjusted individually to allow optimal differentiation between the calcified plaques and the endoluminal contrast media. Carotid stenosis grade was then measured using the NASCET method (13). This grading provided a ratio of the maximum stenotic narrowing (A) and the diameter of the far distal ICA beyond the stenosis and poststenotic dilation (C), calculated by [1-A/C × 100] (Figure 1).

### Semiautomated Perpendicular ICA Stenosis Area Minimal Caliber Computation

**Figure 2:**
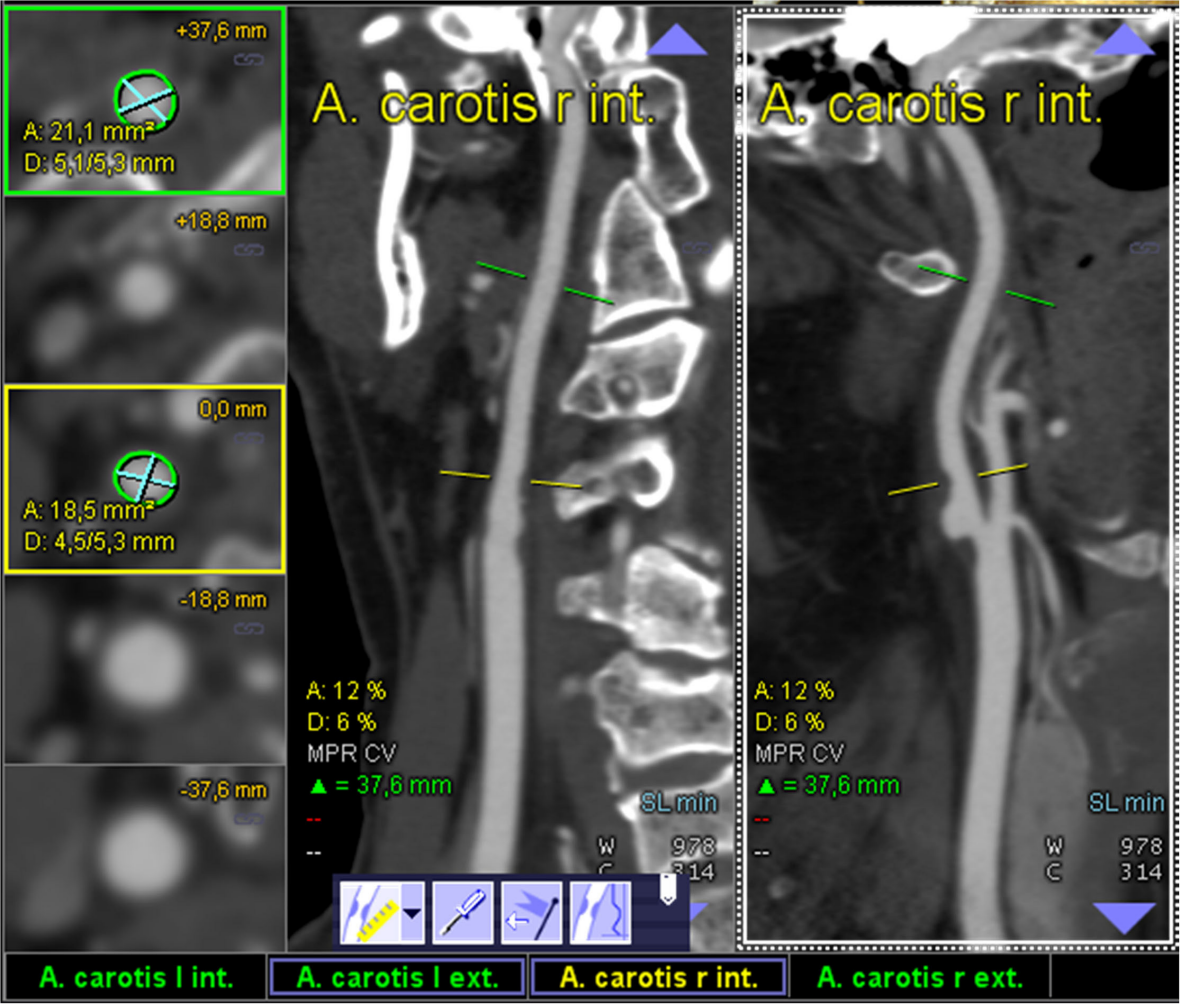
Display of an optimal semiautomated perpendicular stenosis area minimal caliber computation as provided by the “syngo.via” image analysis software: one of the ICAs is automatically detected and the sufficiently contrasted CTA data are displayed as multiplanar reformatted coronal and sagittal view (middle and left column; centerline faded). The segmentally determined luminal area and the underlying non-perpendicular maximum calibers are displayed in the left column (digital caliper).

**Figure 3:**
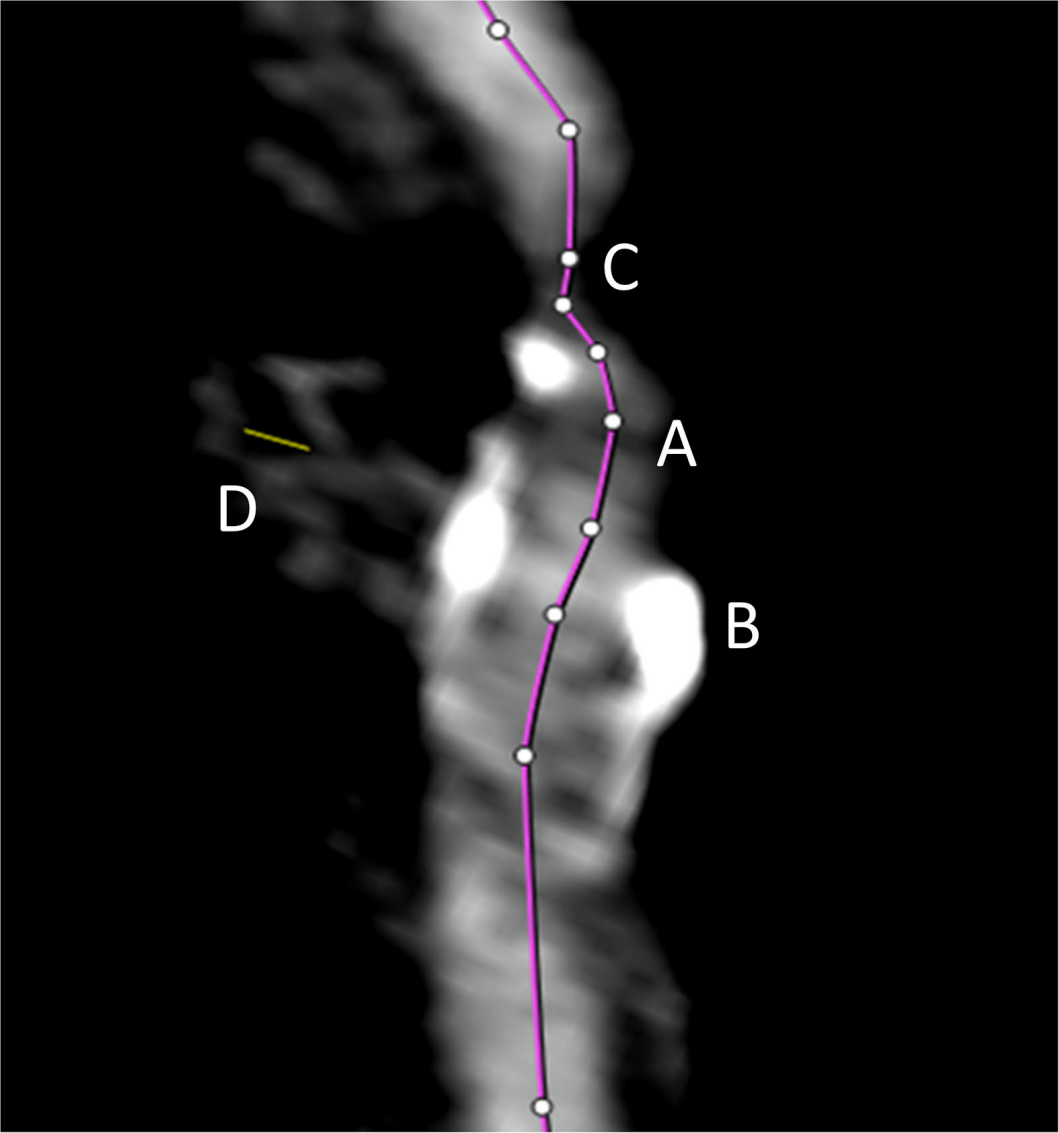
The automated vessel centerline fails in case of insufficient luminal contrast **(A)**, the presence of extensive calcification **(B)**, pseudo-occlusion of the vessel caused by stenotic circulation decrement **(C)**, and in case of accidentally equally contrasted veins in striking distance to the artery **(D)**. In such case, the centerline (purple line) needs to be adjusted manually.

All semiautomated analyses were performed using an image analysis software (syngo.via, Siemens Healthineers, Forchheim, Germany, version VA30A) after preprocessing of the images including contrast optimization and determination of landmarks as previously described (14). This method automatically defined bilateral carotid artery centerlines and provided curved vessel reconstructions. Additional manual adjustments of these centerlines became necessary in case of severe pathologic changes of the stenotic segment, or data quality restrictions. Within the vessel reconstruction, the stenosis was localized and the minimum perpendicular caliber was determined. A second caliber was determined within the closest distal normal appearing ICA segment serving as the reference. The percentage of the stenosis area was finally computed (Figure 2). The center line needed to be adjusted if automatic segmentation of the target vessel failed because of insufficient luminal contrast, extensive calcification, pseudo-occlusion due to stenotic circulation decrement or accidentally in equal measure contrasted veins in proximate distance to the ICA. This was achieved manually by defining the start and end points of the vessel segmentation (Figure 3). The applied contouring algorithm was based on active contour models excluding calcification.

### Statistical Analysis

Continuous and non-continuous variables were presented as median [interquartile range (IQR)] and percentage where appropriate. Stenosis measurements were grouped into clinically relevant NASCET strata (normal, 1–49%, 50–69%, 70–99%, and occlusion) and cross classified by the assessment method. The chi-squared test was applied to determine the differences in proportion of NASCET strata identified by both measurement methods. Diagnostic agreement between both methodologies was further evaluated using Bland–Altman method with calculation of the mean difference (i.e., bias) and the 95% limits of agreement [i.e., mean ± 1.96 standard deviation (SD)] (15).

We assessed interrater reliability and intrarater reliability of both manual and semiautomated NASCET-type caliber measurements calculating the two-way mixed intraclass correlation coefficient (ICC) for absolute agreement, where ICC values <0.40 were considered poor, 0.40–0.59 fair, 0.60–0.74 good, and 0.75–1.00 excellent (16). For this purpose, 20 randomly selected carotid arteries of varying degrees of the disease were independently reassessed by the same rater (TF) after 3 months, and the initial results were compared with the assessments of a blinded expert neuroradiologist (HHK). Significance level was set at 0.05. Statistical analyses were performed with STATA software (version 12.1, StataCorp LLC, College Station, TX, USA) and MedCalc® Statistical Software (version 19.8, MedCalc Software Ltd, Ostend, Belgium).

## Results

### Study Population

During the 12-month study period, we registered 346 consecutive patients with acute cerebral ischemia who underwent CTA acquisition in our academic stroke center. Sixteen patients were excluded due to non-assessable image data (i.e., artifacts from patient motion or dental implants) or non-availability of data. The final study cohort consisted of 330 patients (transient ischemic attack, *n* = 60; acute ischemic stroke, *n* = 270). Upon reviewing the CTA images, 13 arteries were found to have no counterpart vessel due to insufficient imaging quality, leaving 647 carotid artery pairs available for the final comparative analysis. The median age of the patient sample was 74 (IQR, 66–80; range, 38–92) years, 58% were men and 42% women. The median NIHSS score was 4 (IQR, 1–9; range, 0–32) points.

Manual MPR-based diameter measurements found 1–49% stenosis in 143, 50–69% stenosis in 29, 70–99% stenosis in 6, and occlusion in 34 internal carotid arteries, while semiautomated perpendicular minimal stenosis caliber analysis detected 1–49% stenosis in 93, 50–69% stenosis in 27, 70–99% stenosis in 14, and occlusion in 34 internal carotid arteries (*p* = 0.003). Table 1 displays the entire range of diseases identified by both methods.

**Table 1:** Steno-occlusive disease of the ICA by NASCET categories as identified by manual and semiautomated measurements.

		**Semiautomated**, ***n***					
		**0%**	**1–49%**	**50–69%**	**70–99%**	**100%**	**Total**
Manual, *n*	0%	431	4	–	–	–	435
	1–49%	46	83	13	1	–	143
	50–69%	2	6	12	9	–	29
	70–99%	–	–	2	4	–	6
	100%	–	–	–	–	34	34
	Total	479	93	27	14	34	647

*N, number*.

### Diagnostic Agreement Between Manual and Semiautomated Analysis

**Figure 4:**
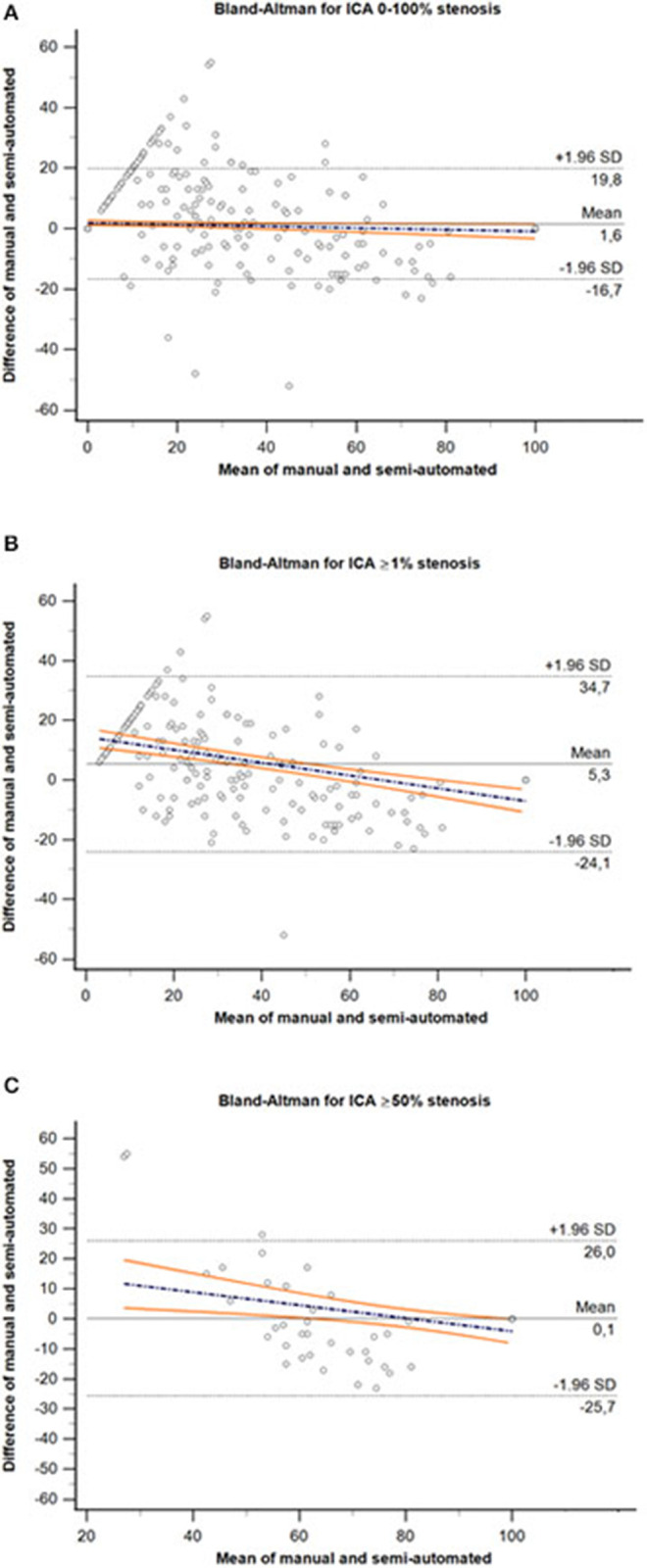
Bland–Altman plot for comparison of the entire range of ICA stenosis **(A)**, ≥1% stenosis **(B)** and ≥50% stenosis **(C)** derived by manual MPR-based diameter measurements and semiautomated minimal caliber computation methods. The dashed black lines represent the upper and lower 95% (1.96 SD) limits of agreement, and solid black line represents the mean difference between both assessment methods. Colored lines represent the regression line of differences including 95% CI. ICA, internal carotid artery; SD, standard deviation.

The Bland–Altman plots are shown in Figure 4. On average, the degree in stenosis detected by semiautomated computation was 1.55% lower than the degree detected by manual MPR diameter measurements. However, the lower and upper 95% (1.96 SD) limits of agreement were −16.7–19.8%, indicating a relatively wide agreement interval between both assessment methods (Figure 4A). A total of 6.04% (52/647) of the data were outside these limits. The repeated Bland–Altman analysis restricted to vessel pairs whose manual measurements resulted in any (≥1%) or clinically relevant (≥50%) stenosis, revealed an even wider agreement interval (mean 5.3%, −24.1–34.7%; mean 0.1%; −25.7–26.0%, respectively) (Figures 4B,C).

### Intrarater Reliability and Interrater Reliability for Manual Diameter and Semiautomated Caliber Measurements, Feasibility

With regard to manual measurements on CTA, we found excellent reliability between the repeated assessments of one expert reader (ICC = 0.997; 95% CI, 0.993–0.999) and between the assessments of two expert readers (ICC = 0.972; 95% CI, 0.936–0.988). For the semiautomated vessel analysis software, both intrarater reliability and interrater reliability were found to be similarly strong (ICC = 0.981; 95% CI, 0.952–0.992 and ICC = 0.745; 95% CI, 0.486–0.883, respectively).

## Discussion

The major finding of this study is that semiautomated perpendicular area minimal caliber computation of ICA stenosis on CTA in patients with cerebral ischemia is feasible for stenosis grading with adjustment for intraluminal stenosis irregularity but holds a limited diagnostic agreement with the widely used technique of MPR-based stenosis diameter measurement. The capacity of semiautomated image analysis algorithms to evaluate the degree of stenosis in the ICA has been recently investigated with heterogeneous results. A method comparison study in 40 patients with known or suspected carotid artery stenosis revealed that software-assisted semi automated stenosis grading achieved high reproducibility, even when inexperienced raters performed the analysis (12). This observation and also another method comparison study reporting a 55% decrease in time to diagnosis of semiautomated compared with conventional CTA-based stenosis grading supports the feasibility and practical utility of semiautomated stenosis grading (13). However, these studies were limited by relatively small sample sizes and uncertain external validity. In our study including 647 carotid artery pairs, we observed diagnostic agreement between independent raters further supporting the practical value of the technique. This observation also supports the feasibility of CT angiography manual multiplanar vessel diameter when applied in the setting of a tertiary stroke center. However, our analysis revealed insufficient diagnostic agreement between manual MPR-based diameter measurement and semiautomated assessment. As semiautomated area computation-based minimal caliber measurement but not manual diameter assessment accounts for variability in stenosis morphology, our observation could suggest underestimation of the degree of stenosis in the more severe and overestimation in mild ranges on manual MPR-based grading. However, absence of a direct anatomical reference technique of stenosis assessment, that is, histopathological evaluation, we cannot rule out overestimation of stenosis in the more severe ranges and underestimation in mild-grade steno-occlusive disease on semiautomated evaluation. This observation substantiates a relevant research gap because limited precision in stenosis grading could bear the danger of misguiding decision-making with respect to revascularization therapies.

Another study in 45 patients with carotid artery stenosis observed a strong correlation (Spearman's rho = 0.975) between manual stenosis grading and software-assisted quantification and even observed that agreement between repeated measures is higher in semiautomated assessment (Spearman's rho = 0.9879) than in manual analysis (Spearman's rho = 0.943). This study even found an improved concordance between repeated measures using software-assisted analysis compared with manual evaluation (17). Our observation of low diagnostic agreement based on the Bland–Altman analysis in a large cohort of patients with cerebral ischemia also suggests that MPR-based diameter measurements and semiautomated area minimal caliber computation assessment methods cannot be used interchangeably (18). When compared to duplex ultrasound-based ICA stenosis grading using German Society for Ultrasound in Medicine criteria or University of Washington stenosis criteria, semiautomated stenosis area computation on CTA displayed only moderate diagnostic agreement with a standard deviation of stenosis differences of more than 20% (19). This observation lends further support to the limited interchangeability of software-assisted ICA stenosis area computation on CTA with other clinically established diagnostic analysis techniques.

In a previous analysis of our carotid artery stenosis cohort, we assessed the agreement between manual stenosis grading on CTA with a multiparametric ultrasound-based grading introduced by The German Society of Ultrasound in Medicine. In this study, the ultrasound-based grading showed overall good agreement with CTA-based grading but Bland–Altman analysis demonstrated widely varying differences between both techniques (20). These observations underscored the need for comparative research on techniques to grade ICA stenosis and provided a detailed characterization of our cohort using standardized ultrasound assessment. Several methodological pitfalls need to be accounted for when using semiautomated stenosis area computation for ICA stenosis grading. Localization of the automated vessel centerline can be compromised by insufficient luminal contrast, extensive calcification, and pseudo-occlusion due to stenotic circulation decrement (21). These sources of error substantiate the necessity of supervision of computational methods by an experienced neuroradiologist and might explain heterogeneity in results of previous studies of software-assisted stenosis grading.

Strengths of our study are detailed analyses of diagnostic agreement, the relatively large sample of arteries assessed, the use of standardized processing protocols, and a complete dataset. Although our study is limited by the absence of histopathology as a reference standard or digital subtraction arteriography as a surrogate reference standard, the availability of complete CTA scans of all arteries included allowed detailed comparison between CTA-based techniques stenosis grading.

The observations derived from this study are based on the use of the software package “syngo.via.” Therefore, we cannot comment on the diagnostic agreement of manual ICA stenosis grading on CTA with semiautomated analysis using other software packages. However, along the progression of our study, we did not change the version of the software package used for analysis, supporting the internal validity of our findings. We cannot rule out rater bias including a possible central tendency bias. However, we noted agreement between independent blinded raters with expertise in cerebrovascular imaging supporting the internal validity of our data. Whereas our study supports the practical and diagnostic value of semiautomated ICA stenosis grading performed in a tertiary stroke center, we cannot comment on the feasibility of its use in the hyperacute assessment of patients with stroke as our image analyses were performed in CTA images that had been obtained prior to the study.

### Conclusion

Our study suggests that MPR-based diameter measurements and semiautomated perpendicular area minimal caliber computation cannot be used interchangeably for the quantification of ICA luminal constriction in steno-occlusive disease in patients with cerebral ischemia. This result demands methodological homogeneity in institutional procedure standards. Beyond that, it is clinically relevant, since a reproducible determination of the degree of stenosis is a critical prerequisite of safe and effective clinical management including decision-making on the indication for revascularization therapy.

## Data Availability Statement

The raw data supporting the conclusions of this article will be made available by the authors, without undue reservation.

## Ethics Statement

The studies involving human participants were reviewed and approved by Ethikkommission an der Technischen Universität Dresden. Written informed consent for participation was not required for this study in accordance with the national legislation and the institutional requirements.

## Author Contributions

TS: supervision of data acquisition, drafting of the manuscript, study concept and design, and interpretation of data. KB: supervision of data acquisition, study concept and design, analysis of data, interpretation of data, and revising manuscript for content. TF: acquisition of data (major role), revising manuscript for content, and interpretation of data. JB and L-PP: revising manuscript for content and interpretation of data. VP: revising manuscript for content, study concept, and interpretation of data. HK: acquisition of data (major role), study concept, revising manuscript for content, and interpretation of data. All authors contributed to the article and approved the submitted version.

## Funding

TS received grants from the Federal German Ministry of Health and the Kurt Goldstein Institute that were not related to this study.

## Conflict of Interest

The authors declare that the research was conducted in the absence of any commercial or financial relationships that could be construed as a potential conflict of interest.

## Publisher's Note

All claims expressed in this article are solely those of the authors and do not necessarily represent those of their affiliated organizations, or those of the publisher, the editors and the reviewers. Any product that may be evaluated in this article, or claim that may be made by its manufacturer, is not guaranteed or endorsed by the publisher.
